# Pyridinium bis­(pyridine-κ*N*)tetra­kis­(thio­cyanato-κ*N*)ferrate(III)

**DOI:** 10.1107/S1600536813011628

**Published:** 2013-05-04

**Authors:** Sergii I. Shylin, Il’ya A. Gural’skiy, Matti Haukka, Anatoliy A. Kapshuk, Elena V. Prisyazhnaya

**Affiliations:** a Department of Chemistry, Taras Shevchenko National University of Kyiv, Volodymyrska 64/13, 01601 Kyiv, Ukraine; b Department of Chemistry, University of Jyväskylä, PO Box 35, FI-40014 Jyväskyä, Finland; c Kyiv National University of Construction and Architecture, Department of Chemistry, Povitroflotsky Avenue 31, 03680 Kyiv, Ukraine

## Abstract

In the title compound, (C_5_H_6_N)[Fe(NCS)_4_(C_5_H_5_N)_2_], the Fe^III^ ion is coordinated by four thio­cyanate N atoms and two pyridine N atoms in a *trans* arrangement, forming an FeN_6_ polyhedron with a slightly distorted octa­hedral geometry. Charge balance is achieved by one pyridinium cation bound to the complex anion *via* N—H⋯S hydrogen bonding. The asymmetric unit consists of one Fe^III^ cation, four thio­cyanate anions, two coordinated pyridine mol­ecules and one pyridinium cation. The structure exhibits π–π inter­actions between pyridine rings [centroid–centroid distances = 3.7267 (2), 3.7811 (2) and 3.8924 (2) Å]. The N atom and a neighboring C atom of the pyridinium cation are statistically disordered with an occupancy ratio of 0.58 (2):0.42 (2).

## Related literature

For the use of materials with mol­ecular assemblies comprising cationic and anionic modules, see: Fritsky *et al.* (1998 ▶, 2004 ▶); Strotmeyer *et al.* (2003 ▶); Kanderal *et al.* (2005 ▶). For Fe^II^–thio­cyanate complexes with aromatic *N*-donor ligands indicating spin crossover, see: Gamez *et al.* (2009 ▶). For related structures, see: Petrusenko *et al.* (1997 ▶); Moroz *et al.* (2010 ▶); Penkova *et al.* (2010 ▶); Shylin *et al.* (2013 ▶).
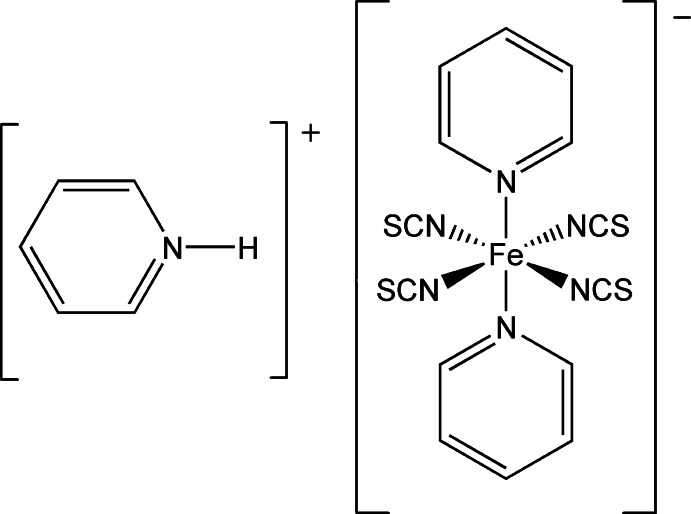


## Experimental

### 

#### Crystal data

(C_5_H_6_N)[Fe(NCS)_4_(C_5_H_5_N)_2_]
*M* *_r_* = 526.48
Monoclinic, 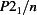

*a* = 10.7650 (7) Å
*b* = 14.0424 (8) Å
*c* = 15.7266 (9) Å
β = 103.244 (3)°
*V* = 2314.1 (2) Å^3^
*Z* = 4
Mo *K*α radiation
μ = 1.03 mm^−1^
*T* = 120 K
0.21 × 0.14 × 0.07 mm

#### Data collection

Bruker Kappa APEXII DUO CCD diffractometer
Absorption correction: multi-scan (*SADABS*; Bruker, 2001 ▶) *T*_min_ = 0.814, *T*_max_ = 0.929
17377 measured reflections
4739 independent reflections
3027 reflections with *I* > 2σ(*I*)
*R*_int_ = 0.065

#### Refinement

*R*[*F* ^2^ > 2σ(*F* ^2^)] = 0.035
*wR*(*F* ^2^) = 0.065
*S* = 0.92
4739 reflections
281 parameters
H-atom parameters constrained
Δρ_max_ = 0.33 e Å^−3^
Δρ_min_ = −0.36 e Å^−3^

### 

Data collection: *APEX2* (Bruker, 2007 ▶); cell refinement: *SAINT* (Bruker, 2007 ▶); data reduction: *SAINT*; program(s) used to solve structure: *SUPERFLIP* (Palatinus & Chapuis, 2007 ▶); program(s) used to refine structure: *SHELXL97* (Sheldrick, 2008 ▶); molecular graphics: *DIAMOND* (Brandenburg, 1997 ▶); software used to prepare material for publication: *SHELXL97*.

## Supplementary Material

Crystal structure: contains datablock(s) I, global. DOI: 10.1107/S1600536813011628/xu5698sup1.cif

Structure factors: contains datablock(s) I. DOI: 10.1107/S1600536813011628/xu5698Isup2.hkl

Supplementary material file. DOI: 10.1107/S1600536813011628/xu5698Isup3.cdx

Additional supplementary materials:  crystallographic information; 3D view; checkCIF report

## Figures and Tables

**Table 1:** Selected bond lengths (Å)

Fe1—N1	2.1591 (18)
Fe1—N2	2.1727 (19)
Fe1—N3	2.012 (2)
Fe1—N4	2.026 (2)
Fe1—N5	2.049 (2)
Fe1—N6	2.034 (2)

**Table 2:** Hydrogen-bond geometry (Å, °)

*D*—H⋯*A*	*D*—H	H⋯*A*	*D*⋯*A*	*D*—H⋯*A*
N7—H7*A*⋯S3^i^	0.88	2.82	3.532 (2)	139
N7—H7*A*⋯S2	0.88	2.86	3.462 (2)	127
N7*A*—H7*AA*⋯S4^ii^	0.88	2.81	3.558 (2)	144
N7*A*—H7*AA*⋯S2	0.88	2.94	3.504 (2)	124

Symmetry codes: (i) 
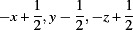
; (ii) 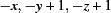.

**Fig. 1.**
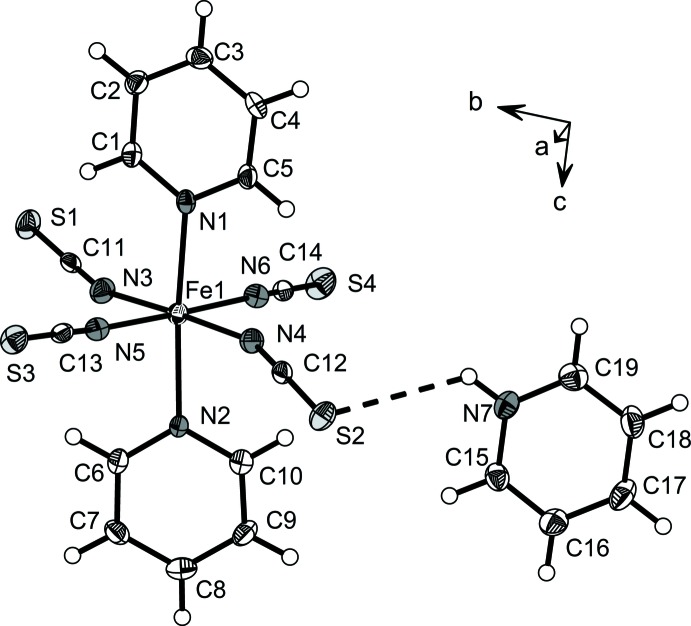
Crystal structure of the title compound with labeling and displacement ellipsoids drawn at the 50% probability level.

**Fig. 2.**
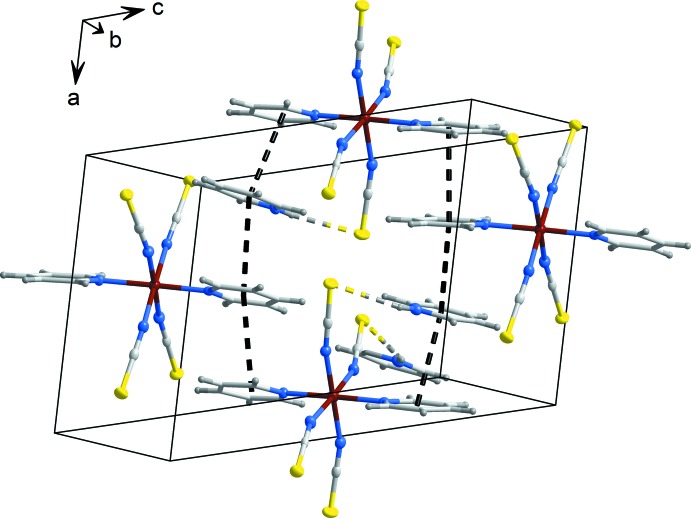
Crystal structure of the title compound showing hydrogen bonds and π–π contacts as dashed lines (carmine = Fe, yellow = S, blue = N, light-grey = C, grey = H).

## supplementary crystallographic information

### Comment

Molecular assemblies consisting of cationic and anionic modules are of great interest for crystal engineering and molecular magnetism (Strotmeyer *et al.*, 2003; Fritsky *et al.*, 2004). Target properties of such compounds can be tuned by different types of intermolecular interactions, such as coordination and hydrogen bonds, *π–π* and lone pair – *π* contacts, *etc*. (Fritsky *et al.*, 1998; Kanderal *et al.*, 2005). In certain cases, even the existence of spin crossover in these complexes can be observed. Therefore, Fe^II^ isothiocyanate complexes with aromatic N-donor ligands attract much attention considering the possible metal ion spin state modulation by variation of a ligand (Gamez *et al.*, 2009). Herein, we attempted to synthesize Fe^II^ thiocyanate complex with 1,5-naphthyridine, however, the reaction of it and [Fe^II^(NCS)_2_(py)_4_] (py = pyridine) in CHCl_3_ in air led to the oxidation of Fe^II^ and to the formation of the title compound.

The compound consists of complex anion [Fe(NCS)_4_(py)_2_]^-^ and pyridinium cation the N7 atom of which is disordered over two alternative sites with the occupancy ratio of 0.58 (2): 0.42 (2). The Fe^III^ ion is sixfold coordinated by four N atoms of thiocyanate anions forming the equatorial plane and two N atoms of two pyridine ligands occupying axial positions (Fig. 1). The distances between Fe^III^ ion and N atoms of the thiocyanate anions are shorter than those between Fe^III^ and N atoms of the pyridine ligands (Table 1), hence FeN_6_ octahedron is slightly distorted. A similar distortion of the coordination polyhedron was reported for the related compound (Hpy)[Fe(NCS)_4_(py)_2_]_._4(cnpz).(py), where cnpz = pyrazine-2-carbonitrile (Shylin *et al.*, 2013). The thiocyanate ligands are only bound through N atoms and are quasi-linear, while the Fe–NCS linkages are bent [Fe1—N3—C11 = 162.43 (19)°, Fe1—N4—C12 = 161.67 (19)°, Fe1—N5—C13 = 165.7 (2)°, Fe1—N6—C14 = 158.7 (2)°]. These structural features are typical for the complexes where the NCS group is N-bound (Petrusenko *et al.*, 1997). The C—N and C—C bond lengths in the coordinated pyridine ligands are normal and close to the values observed in the related structures (Moroz *et al.*, 2010; Penkova *et al.*, 2010).

In the title compound pyridine ligands and pyridinium cations interact with one another *via π–π* stacking, with distances between the centroids of 3.7267 (2), 3.7811 (2) and 3.8924 (2) Å (Fig. 2). Pyridinium cations are also bound to the anionic complex through a number of N—H···S hydrogen bonds (Table 2).

### Experimental

Crystals of the title compound were obtained by adding 1,5-naphthyridine (26 mg, 0.2 mmol) to tetrakis(pyridine)bis(isothiocyanato)iron(II) [Fe(NCS)_2_(py)_4_] (48.8 mg, 0.1 mmol) in CHCl_3_ (5 ml). The solution was left to evaporate in air. In one day this yielded red crystals that were collected, washed with water and dried in air. Yield is 16 mg (30% relative to Fe).

### Refinement

The N atom of the pyridinium cation was disordered over two alternative sites with the occupancy ratio of 0.58/0.42. Hydrogen atoms were positioned geometrically and constrained to ride on their parent atoms, with C—H = 0.95 and N—H = 0.88 Å, and *U*_iso_ = 1.2 *U*_eq_(C, N).

### Crystal data

**Table:**

(C_5_H_6_N)[Fe(NCS)_4_(C_5_H_5_N)_2_]	*F*(000) = 1076
*M**_r_* = 526.48	*D*_x_ = 1.511 Mg m^−^^3^
Monoclinic, *P*2_1_/*n*	Mo *K*α radiation, λ = 0.71073 Å
Hall symbol: -P 2yn	Cell parameters from 3249 reflections
*a* = 10.7650 (7) Å	θ = 2.5–26.9°
*b* = 14.0424 (8) Å	µ = 1.03 mm^−^^1^
*c* = 15.7266 (9) Å	*T* = 120 K
β = 103.244 (3)°	Block, red
*V* = 2314.1 (2) Å^3^	0.21 × 0.14 × 0.07 mm
*Z* = 4	

### Data collection

**Table:**

Bruker Kappa APEXII DUO CCD diffractometer	4739 independent reflections
Radiation source: fine-focus sealed tube	3027 reflections with *I* > 2σ(*I*)
Curved graphite crystal monochromator	*R*_int_ = 0.065
Detector resolution: 16 pixels mm^-1^	θ_max_ = 26.4°, θ_min_ = 2.0°
φ scans and ω scans with κ offset	*h* = −13→13
Absorption correction: multi-scan (*SADABS*; Bruker, 2001)	*k* = −17→17
*T*_min_ = 0.814, *T*_max_ = 0.929	*l* = −19→16
17377 measured reflections	

### Refinement

**Table:**

Refinement on *F*^2^	Primary atom site location: structure-invariant direct methods
Least-squares matrix: full	Secondary atom site location: difference Fourier map
*R*[*F*^2^ > 2σ(*F*^2^)] = 0.035	Hydrogen site location: inferred from neighbouring sites
*wR*(*F*^2^) = 0.065	H-atom parameters constrained
*S* = 0.92	*w* = 1/[σ^2^(*F*_o_^2^) + (0.024*P*)^2^] where *P* = (*F*_o_^2^ + 2*F*_c_^2^)/3
4739 reflections	(Δ/σ)_max_ = 0.001
281 parameters	Δρ_max_ = 0.33 e Å^−^^3^
0 restraints	Δρ_min_ = −0.36 e Å^−^^3^

### Special details

**Table:**

Geometry. All e.s.d.'s (except the e.s.d. in the dihedral angle between two l.s. planes) are estimated using the full covariance matrix. The cell e.s.d.'s are taken into account individually in the estimation of e.s.d.'s in distances, angles and torsion angles; correlations between e.s.d.'s in cell parameters are only used when they are defined by crystal symmetry. An approximate (isotropic) treatment of cell e.s.d.'s is used for estimating e.s.d.'s involving l.s. planes.
Refinement. Refinement of *F*^2^ against ALL reflections. The weighted *R*-factor *wR* and goodness of fit *S* are based on *F*^2^, conventional *R*-factors *R* are based on *F*, with *F* set to zero for negative *F*^2^. The threshold expression of *F*^2^ > σ(*F*^2^) is used only for calculating *R*-factors(gt) *etc*. and is not relevant to the choice of reflections for refinement. *R*-factors based on *F*^2^ are statistically about twice as large as those based on *F*, and *R*- factors based on ALL data will be even larger.

### Fractional atomic coordinates and isotropic or equivalent isotropic displacement parameters (Å^2^)

**Table:**

	*x*	*y*	*z*	*U*_iso_*/*U*_eq_	Occ. (<1)
Fe1	0.02757 (4)	0.74386 (2)	0.35673 (2)	0.01575 (10)	
S1	−0.26742 (7)	0.99542 (5)	0.35060 (4)	0.02555 (18)	
S2	0.33249 (7)	0.49516 (5)	0.37979 (4)	0.02563 (18)	
S3	0.41585 (7)	0.90657 (5)	0.34609 (4)	0.02646 (18)	
S4	−0.37170 (7)	0.61072 (5)	0.37071 (5)	0.03125 (19)	
N1	−0.02004 (18)	0.75765 (14)	0.21626 (11)	0.0158 (5)	
N2	0.0714 (2)	0.71819 (13)	0.49668 (12)	0.0160 (5)	
N3	−0.0733 (2)	0.86076 (15)	0.37176 (13)	0.0227 (5)	
N4	0.1344 (2)	0.62743 (14)	0.34665 (13)	0.0212 (5)	
N5	0.1848 (2)	0.82818 (14)	0.36327 (12)	0.0204 (5)	
N6	−0.1316 (2)	0.66311 (14)	0.34861 (13)	0.0224 (5)	
N7	0.2005 (2)	0.26981 (16)	0.34397 (14)	0.0236 (7)	0.58 (2)
H7A	0.1943	0.3257	0.3180	0.028*	0.58 (2)
N7A	0.2499 (2)	0.26424 (16)	0.43092 (15)	0.0236 (7)	0.42 (2)
H7AA	0.2757	0.3165	0.4606	0.028*	0.42 (2)
C12	0.2170 (3)	0.57121 (17)	0.36058 (15)	0.0181 (6)	
C14	−0.2322 (3)	0.64138 (17)	0.35776 (15)	0.0185 (6)	
C11	−0.1543 (3)	0.91796 (17)	0.36277 (14)	0.0171 (6)	
C13	0.2810 (3)	0.86161 (17)	0.35567 (15)	0.0183 (6)	
C5	−0.0212 (2)	0.68043 (17)	0.16522 (15)	0.0185 (6)	
H5	0.0013	0.6204	0.1923	0.022*	
C1	−0.0523 (2)	0.84165 (17)	0.17635 (15)	0.0192 (6)	
H1	−0.0526	0.8967	0.2114	0.023*	
C15	0.2499 (2)	0.26424 (16)	0.43092 (15)	0.0236 (7)	0.58 (2)
H15	0.2777	0.3207	0.4630	0.028*	0.58 (2)
C15A	0.2005 (2)	0.26981 (16)	0.34397 (14)	0.0236 (7)	0.42 (2)
H15A	0.1938	0.3301	0.3159	0.028*	0.42 (2)
C6	0.1024 (2)	0.78922 (17)	0.55371 (15)	0.0187 (6)	
H6	0.1088	0.8517	0.5320	0.022*	
C10	0.0651 (3)	0.63017 (18)	0.52842 (16)	0.0220 (6)	
H10	0.0438	0.5788	0.4884	0.026*	
C4	−0.0536 (2)	0.68502 (18)	0.07530 (15)	0.0207 (6)	
H4	−0.0546	0.6290	0.0413	0.025*	
C3	−0.0846 (2)	0.77183 (18)	0.03525 (16)	0.0232 (6)	
H3	−0.1053	0.7768	−0.0266	0.028*	
C8	0.1183 (3)	0.68517 (18)	0.67526 (16)	0.0246 (6)	
H8	0.1336	0.6740	0.7364	0.030*	
C7	0.1258 (3)	0.77584 (17)	0.64337 (15)	0.0227 (6)	
H7	0.1466	0.8283	0.6821	0.027*	
C2	−0.0849 (2)	0.85126 (18)	0.08662 (15)	0.0208 (6)	
H2	−0.1073	0.9118	0.0607	0.025*	
C16	0.2603 (3)	0.18020 (18)	0.47284 (17)	0.0272 (7)	
H16	0.2947	0.1771	0.5341	0.033*	
C19	0.1606 (3)	0.19118 (18)	0.29654 (17)	0.0268 (7)	
H19	0.1259	0.1959	0.2355	0.032*	
C9	0.0882 (3)	0.61079 (18)	0.61679 (16)	0.0245 (7)	
H9	0.0834	0.5474	0.6369	0.029*	
C17	0.2203 (3)	0.09859 (19)	0.42586 (17)	0.0303 (7)	
H17	0.2271	0.0387	0.4546	0.036*	
C18	0.1707 (3)	0.10391 (18)	0.33741 (17)	0.0301 (7)	
H18	0.1436	0.0478	0.3047	0.036*	

### Atomic displacement parameters (Å^2^)

**Table:**

	*U*^11^	*U*^22^	*U*^33^	*U*^12^	*U*^13^	*U*^23^
Fe1	0.01694 (19)	0.01441 (19)	0.01572 (17)	0.00150 (16)	0.00334 (13)	−0.00003 (15)
S1	0.0250 (4)	0.0179 (4)	0.0332 (4)	0.0067 (3)	0.0055 (3)	0.0014 (3)
S2	0.0244 (4)	0.0204 (4)	0.0326 (4)	0.0075 (3)	0.0075 (3)	0.0044 (3)
S3	0.0224 (4)	0.0271 (4)	0.0307 (4)	−0.0042 (3)	0.0077 (3)	0.0048 (3)
S4	0.0240 (4)	0.0326 (4)	0.0386 (4)	−0.0042 (3)	0.0104 (3)	0.0080 (3)
N1	0.0135 (12)	0.0135 (11)	0.0204 (11)	−0.0022 (9)	0.0042 (8)	−0.0012 (9)
N2	0.0197 (13)	0.0120 (11)	0.0163 (10)	−0.0010 (9)	0.0039 (9)	0.0003 (9)
N3	0.0276 (14)	0.0194 (12)	0.0216 (12)	0.0044 (11)	0.0071 (10)	0.0003 (10)
N6	0.0251 (15)	0.0219 (12)	0.0192 (12)	−0.0014 (11)	0.0030 (10)	0.0001 (10)
N5	0.0216 (14)	0.0203 (12)	0.0187 (11)	−0.0004 (10)	0.0033 (9)	0.0013 (9)
N4	0.0239 (14)	0.0186 (12)	0.0209 (12)	0.0031 (11)	0.0048 (10)	−0.0014 (10)
C12	0.0216 (16)	0.0162 (14)	0.0165 (13)	−0.0051 (12)	0.0044 (11)	−0.0002 (11)
C14	0.0257 (18)	0.0139 (14)	0.0145 (13)	0.0025 (12)	0.0014 (12)	−0.0010 (11)
C11	0.0233 (16)	0.0172 (14)	0.0105 (12)	−0.0051 (12)	0.0033 (11)	−0.0020 (10)
C13	0.0258 (17)	0.0151 (13)	0.0133 (13)	0.0056 (13)	0.0026 (11)	0.0011 (11)
C5	0.0186 (15)	0.0141 (13)	0.0223 (14)	−0.0044 (12)	0.0037 (11)	−0.0004 (11)
C1	0.0206 (16)	0.0148 (14)	0.0223 (14)	−0.0033 (12)	0.0055 (11)	−0.0003 (11)
C15	0.0283 (16)	0.0215 (15)	0.0229 (14)	−0.0032 (12)	0.0100 (11)	−0.0042 (11)
N7	0.0261 (15)	0.0195 (14)	0.0273 (15)	0.0031 (11)	0.0102 (11)	0.0026 (11)
N7A	0.0283 (16)	0.0215 (15)	0.0229 (14)	−0.0032 (12)	0.0100 (11)	−0.0042 (11)
C15A	0.0261 (15)	0.0195 (14)	0.0273 (15)	0.0031 (11)	0.0102 (11)	0.0026 (11)
C6	0.0186 (15)	0.0144 (13)	0.0233 (14)	−0.0005 (11)	0.0051 (11)	0.0013 (11)
C10	0.0243 (16)	0.0194 (14)	0.0220 (14)	−0.0008 (12)	0.0047 (12)	0.0002 (12)
C4	0.0211 (16)	0.0198 (15)	0.0209 (14)	−0.0049 (12)	0.0041 (11)	−0.0082 (12)
C3	0.0217 (16)	0.0288 (16)	0.0177 (13)	−0.0087 (12)	0.0014 (11)	0.0024 (12)
C8	0.0242 (17)	0.0326 (16)	0.0171 (13)	0.0022 (13)	0.0047 (12)	0.0040 (13)
C7	0.0249 (16)	0.0229 (15)	0.0189 (14)	−0.0007 (12)	0.0023 (12)	−0.0060 (12)
C2	0.0207 (16)	0.0189 (14)	0.0223 (14)	−0.0019 (12)	0.0036 (12)	0.0037 (12)
C16	0.0354 (19)	0.0279 (16)	0.0213 (14)	0.0020 (14)	0.0125 (13)	0.0014 (13)
C19	0.0294 (18)	0.0295 (16)	0.0231 (14)	0.0027 (14)	0.0094 (12)	−0.0024 (13)
C9	0.0303 (17)	0.0186 (15)	0.0248 (15)	0.0010 (13)	0.0065 (12)	0.0079 (12)
C17	0.044 (2)	0.0209 (16)	0.0305 (16)	0.0029 (14)	0.0187 (14)	0.0070 (13)
C18	0.041 (2)	0.0208 (16)	0.0314 (16)	−0.0046 (14)	0.0131 (14)	−0.0068 (13)

### Geometric parameters (Å, º)

**Table:**

Fe1—N1	2.1591 (18)	C15—H15	0.9500
Fe1—N2	2.1727 (19)	N7—C19	1.346 (3)
Fe1—N3	2.012 (2)	N7—H7A	0.8800
Fe1—N4	2.026 (2)	C6—C7	1.387 (3)
Fe1—N5	2.049 (2)	C6—H6	0.9500
Fe1—N6	2.034 (2)	C10—C9	1.382 (3)
S1—C11	1.611 (3)	C10—H10	0.9500
S2—C12	1.614 (3)	C4—C3	1.377 (3)
S3—C13	1.621 (3)	C4—H4	0.9500
S4—C14	1.620 (3)	C3—C2	1.378 (3)
N1—C1	1.344 (3)	C3—H3	0.9500
N1—C5	1.347 (3)	C8—C7	1.378 (3)
N2—C6	1.332 (3)	C8—C9	1.380 (3)
N2—C10	1.341 (3)	C8—H8	0.9500
N3—C11	1.170 (3)	C7—H7	0.9500
N6—C14	1.165 (3)	C2—H2	0.9500
N5—C13	1.168 (3)	C16—C17	1.378 (3)
N4—C12	1.172 (3)	C16—H16	0.9500
C5—C4	1.378 (3)	C19—C18	1.377 (3)
C5—H5	0.9500	C19—H19	0.9500
C1—C2	1.380 (3)	C9—H9	0.9500
C1—H1	0.9500	C17—C18	1.373 (4)
C15—C16	1.344 (3)	C17—H17	0.9500
C15—N7	1.351 (3)	C18—H18	0.9500
			
N3—Fe1—N4	177.50 (9)	C19—N7—H7A	119.5
N3—Fe1—N6	89.58 (9)	C15—N7—H7A	119.5
N4—Fe1—N6	91.74 (9)	N2—C6—C7	122.8 (2)
N3—Fe1—N5	89.11 (9)	N2—C6—H6	118.6
N4—Fe1—N5	89.60 (9)	C7—C6—H6	118.6
N6—Fe1—N5	178.43 (9)	N2—C10—C9	122.9 (2)
N3—Fe1—N1	92.24 (8)	N2—C10—H10	118.5
N4—Fe1—N1	89.90 (8)	C9—C10—H10	118.5
N6—Fe1—N1	89.24 (8)	C3—C4—C5	119.2 (2)
N5—Fe1—N1	89.96 (8)	C3—C4—H4	120.4
N3—Fe1—N2	90.81 (8)	C5—C4—H4	120.4
N4—Fe1—N2	87.13 (8)	C4—C3—C2	118.8 (2)
N6—Fe1—N2	87.45 (8)	C4—C3—H3	120.6
N5—Fe1—N2	93.43 (8)	C2—C3—H3	120.6
N1—Fe1—N2	175.48 (7)	C7—C8—C9	118.8 (2)
C1—N1—C5	117.51 (19)	C7—C8—H8	120.6
C1—N1—Fe1	122.14 (16)	C9—C8—H8	120.6
C5—N1—Fe1	120.33 (15)	C8—C7—C6	118.9 (2)
C6—N2—C10	117.8 (2)	C8—C7—H7	120.6
C6—N2—Fe1	121.38 (15)	C6—C7—H7	120.6
C10—N2—Fe1	120.83 (15)	C3—C2—C1	119.1 (2)
C11—N3—Fe1	162.43 (19)	C3—C2—H2	120.5
C14—N6—Fe1	158.7 (2)	C1—C2—H2	120.5
C13—N5—Fe1	165.7 (2)	C15—C16—C17	119.1 (2)
C12—N4—Fe1	161.67 (19)	C15—C16—H16	120.4
N4—C12—S2	179.0 (2)	C17—C16—H16	120.4
N6—C14—S4	179.7 (3)	N7—C19—C18	119.4 (2)
N3—C11—S1	179.1 (2)	N7—C19—H19	120.3
N5—C13—S3	179.0 (2)	C18—C19—H19	120.3
N1—C5—C4	122.6 (2)	C8—C9—C10	118.8 (2)
N1—C5—H5	118.7	C8—C9—H9	120.6
C4—C5—H5	118.7	C10—C9—H9	120.6
N1—C1—C2	122.8 (2)	C18—C17—C16	119.9 (2)
N1—C1—H1	118.6	C18—C17—H17	120.0
C2—C1—H1	118.6	C16—C17—H17	120.0
C16—C15—N7	121.1 (2)	C17—C18—C19	119.5 (2)
C16—C15—H15	119.4	C17—C18—H18	120.3
N7—C15—H15	119.4	C19—C18—H18	120.3
C19—N7—C15	121.0 (2)		

### Hydrogen-bond geometry (Å, º)

**Table:**

*D*—H···*A*	*D*—H	H···*A*	*D*···*A*	*D*—H···*A*
N7—H7*A*···S3^i^	0.88	2.82	3.532 (2)	139
N7—H7*A*···S2	0.88	2.86	3.462 (2)	127
N7*A*—H7*AA*···S4^ii^	0.88	2.81	3.558 (2)	144
N7*A*—H7*AA*···S2	0.88	2.94	3.504 (2)	124

Symmetry codes: (i) −*x*+1/2, *y*−1/2, −*z*+1/2; (ii) −*x*, −*y*+1, −*z*+1.
